# Advances in classification-based treatment of Kümmell disease

**DOI:** 10.3389/fsurg.2026.1987453

**Published:** 2026-10-30

**Authors:** Zhou Zi'an, Wu Feipeng, Buweimaieryemu Kudereti, Li Dapeng

**Affiliations:** 1 Department of Spine Surgery, Affiliated Hospital of Jiangsu University, Zhenjiang, Jiangsu, China; 2 School of Medicine, Jiangsu University, Zhenjiang, Jiangsu, China; 3 The Third Clinical Medical College of Ningxia Medical University, Yinchuan, Ningxia, China

**Keywords:** intravertebral cleft, Kümmell disease, minimally invasive double-anchoring technique, osteoporotic vertebral compression fracture, percutaneous kyphoplasty

## Abstract

**Background:**

Kümmell disease is a special type of delayed vertebral collapse after osteoporotic vertebral compression fracture, characterized by vertebral ischemic osteonecrosis, intravertebral cleft, fracture nonunion, and pseudoarthrosis-like instability. Its treatment should shift from simple pain relief to classification-guided stability reconstruction.

**Methods:**

This narrative review was conducted with a structured literature search focusing on the pathological mechanism, imaging classification, vertebral augmentation, bone cement distribution, anchoring techniques, fixation strategies, and complication management of Kümmell disease.

**Main findings:**

Classification-based treatment is important because patients differ in vertebral collapse, intravertebral cleft morphology, posterior wall integrity, dynamic instability, spinal canal compromise, and neurological status. Stable or early-stage cases may be treated with conservative management, percutaneous vertebroplasty (PVP), or percutaneous kyphoplasty (PKP). Unstable cases with obvious intravertebral clefts, cement aggregation, or high risk of cement loosening and migration may require bone cement distribution optimization, anchoring techniques, bone-filling mesh containers, or intravertebral support devices. Advanced or complex cases with severe posterior wall defect, severe spinal canal compromise, neurological deficit, rigid kyphotic deformity, or poor reducibility may require posterior fixation, decompression, or deformity correction. The treatment boundary for stage III patients with posterior wall disruption but without neurological symptoms remains controversial and requires individualized decision-making.

**Conclusions:**

Kümmell disease treatment should shift toward classification-guided decision-making, vertebral stability reconstruction, and whole-process osteoporosis management. Emerging techniques may provide additional options for selected patients, but current evidence remains limited and further multicenter prospective studies are needed

## 1 Introduction

Osteoporotic vertebral compression fracture is a common spinal disease in the elderly. Most patients can achieve pain relief after conservative treatment or vertebral augmentation. However, some patients experience a brief asymptomatic period after minor trauma, followed by delayed vertebral collapse, persistent low back pain, local kyphotic deformity and even neurological dysfunction. These lesion-like conditions are usually classified as Kümmell disease (1, 2). Unlike ordinary acute osteoporotic vertebral compression fractures, the core problem of Kümmell disease is not the simple loss of vertebral height, but the dynamic instability of the affected vertebra caused by vertebral ischemic osteonecrosis, intravertebral cleft, fracture nonunion and pseudoarthrosis-like instability (3, 4). Therefore, its treatment goal should not only focus on short-term pain relief, but also pay attention to the reconstruction of vertebral stability, kyphotic deformity control, prevention of bone cement migration and neurological function protection.

At present, the treatment methods of Kümmell disease include conservative treatment, percutaneous vertebroplasty (PVP), percutaneous kyphoplasty (PKP), bone cement distribution optimization, pedicle anchoring, instrumentation augmentation and decompression procedures, etc. There are obvious differences in the morphology of intravertebral clefts, posterior wall integrity, degree of dynamic instability and neurological compression among patients with different classifications, which determines that the treatment strategy should not simply apply the ordinary vertebral compression fracture model. Especially for patients with stage III Kümmell disease and posterior wall disruption but without neurological symptoms, the choice of simple vertebral augmentation, composite anchoring techniques or combined internal fixation remains controversial (5). Based on this, this paper reviews the pathological basis of Kümmell disease, imaging classification and PVP, PKP, bone cement distribution optimization, minimally invasive double-anchoring technique and stabilization reconstruction of complex cases from the perspective of classification evaluation to guide treatment selection.

## 2 Literature search strategy and selection criteria

This review was designed as a narrative review with a structured literature search. The literature search strategy and selection criteria are summarized in Table 1. The literature search was performed in PubMed, Web of Science, Embase, CNKI, WanFang, and Google Scholar from database inception to September 2026. The following search terms were used alone or in combination: “Kümmell disease”, “Kummell disease”, “osteoporotic vertebral compression fracture”, “delayed vertebral collapse”, “intravertebral cleft”, “intravertebral vacuum cleft”, “vertebral osteonecrosis”, “percutaneous vertebroplasty”, “percutaneous kyphoplasty”, “bone cement leakage”, “bone cement migration”, “pedicle screw fixation”, “bone cement-augmented pedicle screw”, “bone-filling mesh container”, “intravertebral support device”, and “double-anchoring technique”.

**Table 1:** Summary of literature search and selection strategy.

Item	Description
Review type	Narrative review with structured literature search
Databases	PubMed, Web of Science, Embase, CNKI, WanFang, Google Scholar
Search period	From database inception to September 2026
Main search terms	“Kümmell disease”， “Kummell disease”， “osteoporotic vertebral compression fracture”， “intravertebral cleft”， “percutaneous vertebroplasty”， “percutaneous kyphoplasty”， “bone cement leakage”， “bone cement migration”， “pedicle screw fixation”， “double-anchoring technique”
Inclusion criteria	Studies related to the pathology, imaging classification, treatment strategies, minimally invasive techniques, fixation/decompression strategies, or complications of Kümmell disease
Exclusion criteria	Duplicate studies, irrelevant studies, studies without sufficient clinical or imaging information, studies on ordinary osteoporotic vertebral compression fractures without delayed collapse or intravertebral cleft, and articles without available full text
Screening process	Title and abstract screening followed by full-text review; additional studies identified through reference lists
Evidence consideration	Study design, sample size, follow-up duration, consistency of findings, and clinical relevance were considered; no formal risk-of-bias scoring was performed because this was not a systematic review

Studies were considered eligible if they were related to the pathological mechanism, imaging features, classification systems, vertebral augmentation, bone cement distribution, anchoring techniques, fixation and decompression strategies, or complication management of Kümmell disease. Original clinical studies, retrospective comparative studies, systematic reviews, meta-analyses, case series, technical reports, and biomechanical studies were considered. Studies were excluded if they were not related to Kümmell disease, focused on ordinary osteoporotic vertebral compression fractures without delayed collapse or intravertebral cleft, lacked sufficient clinical or imaging information, were duplicate publications, or were not available in full text.

The initial screening was performed according to the title and abstract. Potentially relevant studies were then reviewed in full text. Additional studies were identified by manually checking the reference lists of the included articles. Because this article is a narrative review rather than a systematic review, no formal risk-of-bias scoring was performed. However, the strength of evidence was considered according to study design, sample size, follow-up duration, consistency of findings, and clinical relevance. Systematic reviews, meta-analyses, and comparative clinical studies were given greater weight than small case series or preliminary technical reports. For emerging techniques, such as minimally invasive double-anchoring techniques and intravertebral support devices, the limited level of evidence was explicitly considered when interpreting their clinical value.

## 3 Pathological basis and imaging classification

Kümmell disease was originally used to describe a type of disease in which a period of relative asymptomatic status occurred after a minor spinal injury, followed by delayed vertebral collapse, painful kyphosis and dysfunction. With the deepening of imaging and pathological understanding, its concept has gradually expanded from “post-traumatic delayed vertebral collapse” to the comprehensive manifestations of vertebral ischemic osteonecrosis, fracture nonunion and intravertebral instability after osteoporotic vertebral compression fracture (1–3). Therefore, Kümmell disease is not a natural continuation of ordinary osteoporotic vertebral compression fractures, but a special type of lesion with the characteristics of delayed development, osteonecrosis and dynamic instability.

Ischemic osteonecrosis of the vertebral body is an important basis for the development of Kümmell disease. The strength of the vertebral bone of elderly osteoporosis patients decreases, and slight external force can cause microcirculation damage in the endplate, cancellous bone and vertebrae; after local blood supply disorders, the repair ability of the fracture area decreases, and it is difficult to form effective bone healing between the necrotic bone and the normal bone. The affected vertebra repeatedly undergoes micromotion under loading and positional changes, and gradually develops height loss, wedge deformity and local kyphotic deformity (2, 6). Intravertebral cleft sign is one of the most representative imaging manifestations of Kümmell disease, which can be shown as intravertebral vacuum signs on x-ray or CT, and fluid signs on MRI. Its significance is not limited to diagnosis, but often suggests that there are necrotic cavities, fracture nonunion and pseudoarthrosis-like activity in the vertebral body (4, 6, 7). The size, position and morphology of the cleft cavity and its relationship with the posterior wall of the vertebral body directly affect the distribution pattern of bone cement, leakage risk, postoperative stability and risk of recurrent collapse.

Imaging evaluation is the basis of classification-based treatment of Kümmell disease. x-rays can show vertebral body height loss, wedge deformity, local kyphosis and partial vacuum signs. Dynamic radiographs or supine-standing position comparisons can help determine whether there is reducible collapse and dynamic instability in the affected vertebra. CT can clearly show the extent of the intravertebral cleft, endplate destruction, posterior wall integrity, cortical bone defects and bone fragments protruding into the spinal canal, which is an important basis for evaluating the risk of bone cement leakage and designing the puncture pathway. MRI helps identify fluid signs, bone marrow edema, spinal canal compression and neural structure involvement, and can be used to differentiate spinal tumors, infections and ordinary acute osteoporotic vertebral compression fractures.

From the perspective of treatment, Kümmell disease should be classified according to vertebral body height loss, intravertebral cleft, dynamic instability, posterior wall integrity and neurological function status. Classic stage I is usually defined as mild vertebral body height loss, with no obvious collapse on x-ray but necrosis or cleft manifestations on MRI; stage II is characterized by aggravated vertebral collapse accompanied by dynamic activity, but the posterior wall remains relatively intact; stage III involves posterior wall collapse, spinal canal compromise, and even neurological dysfunction (8). Stage I and some stage II patients can choose conservative treatment, PVP or PKP; those who have obvious clefts, pseudoarthrosis-like activity or high risk of bone cement migration should consider composite stabilization techniques such as bone cement distribution optimization, pedicle anchoring or minimally invasive double-anchoring technique; for obvious spinal canal compromise, neurological dysfunction or severe kyphotic deformity, simple vertebral augmentation is usually insufficient, and posterior instrumented fusion, decompression or deformity correction and reconstruction should be further selected. Based on these pathological and imaging features, a classification-guided treatment algorithm for Kümmell disease is summarized in Figure 1.

**Figure 1:**
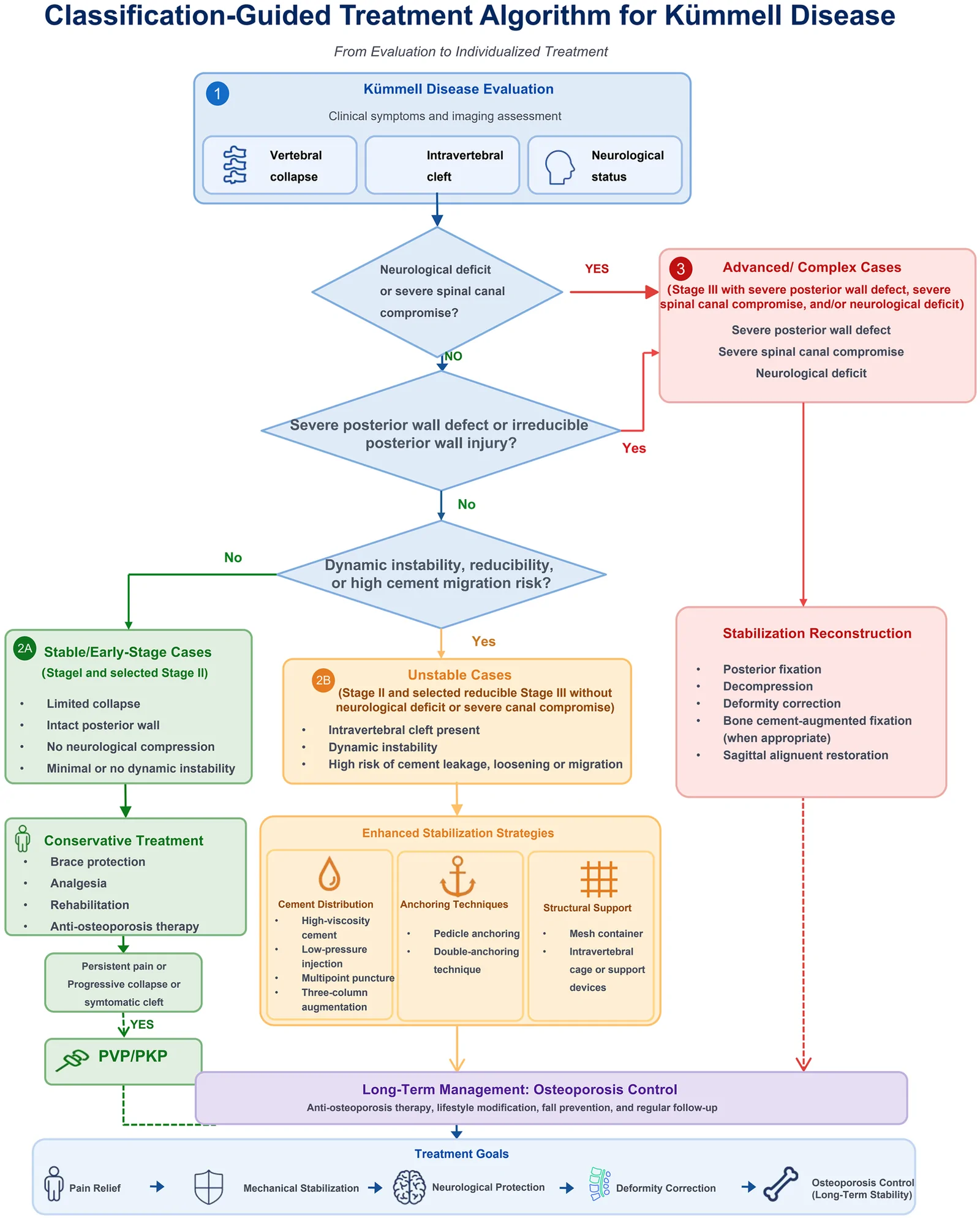
Classification-guided treatment algorithm for kümmell disease. The algorithm summarizes the evaluation of vertebral collapse, intravertebral cleft and neurological status, and stratifies patients into stable/early-stage, unstable and advanced/complex groups according to posterior wall integrity, dynamic stability and neurological involvement. Stable cases may be managed with conservative treatment or PVP/PKP according to symptoms and progression; unstable cases may require cement distribution optimization, anchoring techniques or structural support; advanced cases with severe spinal canal compromise or neurological deficits may require posterior fixation, decompression and deformity correction. Long-term osteoporosis management should be continued throughout follow-up. PVP, percutaneous vertebroplasty; PKP, percutaneous kyphoplasty.

## 4 Basic treatment and vertebral augmentation under classification guidance

The treatment of Kümmell disease should be based on classification evaluation, and the degree of symptoms, vertebral body height loss, cleft morphology, posterior wall integrity, dynamic instability and neurological function status should be comprehensively judged. For stable cases with mild pain, limited vertebral collapse, intact posterior wall, no obvious dynamic instability and neurological compression, conservative management can be adopted first, including short-term bed rest, brace protection, analgesia, functional rehabilitation, fall prevention and anti-osteoporotic therapy. However, Kümmell disease is often accompanied by necrotic cavities and pseudoarthrosis-like activity in the vertebral body. It is difficult to reverse fracture nonunion with simple conservative treatment. If the pain persists, collapse progresses or cleft activity is obvious, surgical evaluation should be performed in time. Before vertebral augmentation, posterior wall integrity and intravertebral cleft morphology should be carefully evaluated, because the risk of bone cement leakage is associated with posterior wall defects, cleft characteristics, bone quality, cement viscosity, injection pressure and injection volume (9). For patients with obvious intravertebral clefts, cement aggregation or a high risk of migration, composite stabilization techniques, including cement distribution optimization, pedicle anchoring, minimally invasive double-anchoring techniques or container-assisted filling, may be considered according to the classification and stability of the lesion (10). Anti-osteoporotic therapy should be carried out throughout the perioperative period and follow-up stage to reduce the risk of recurrent collapse, adjacent vertebral fracture and instrumentation failure; teriparatide may help reduce further vertebral collapse in patients with severe osteoporosis and Kümmell disease (11).

PVP and PKP are still the main minimally invasive methods for patients without obvious neurological symptoms in the early and middle stages. PVP can rapidly enhance vertebral strength, reduce cleft micromotion and relieve pain. It has the advantages of minimal trauma, simple operation, short operative time and low cost. However, in cases with sclerotic cleft walls or large necrotic cavities, bone cement tends to form mass-like filling, and interlocking with surrounding cancellous bone is insufficient, increasing the risks of loosening, migration, recurrent collapse and leakage. PKP involves low-pressure injection of bone cement after balloon expansion, which helps restore partial vertebral height, improve local kyphosis and reduce leakage risk, but the cost and procedural complexity are relatively higher. Previous studies have shown that both PVP and PKP can improve pain and function. PKP has greater advantages in vertebral height restoration, kyphotic correction and leakage control, while PVP has greater advantages in operative time and economic burden (12, 13). Network meta-analysis also suggests that PVP, PKP and bone-filling mesh containers can improve symptoms. Among them, PKP is more advantageous for deformity correction, and bone-filling mesh containers may be safer in reducing leakage (14). Therefore, the choice between the two should not be simply compared, but should be based on classification, posterior wall integrity, cleft morphology and reduction potential: PVP can be selected for stable cases with small clefts and intact posterior walls; PKP can be selected for cases with obvious height loss, increased kyphosis and remaining reduction potential; for cases with obvious clefts, bone cement aggregation or high risk of migration, composite stabilization techniques such as distribution optimization, pedicle anchoring, minimally invasive double-anchoring technique or container-assisted filling should be considered (10).

## 5 New technologies for minimally invasive augmentation and stabilization reconstruction

With the deepening understanding of the mechanism of intravertebral clefts, pseudoarthrosis-like activity and bone cement migration in the vertebral body, the minimally invasive treatment of Kümmell disease has changed from simply “filling the cleft” to “improving distribution, enhancing anchoring, and rebuilding stability”. In cases with large cleft cavities, sclerotic cleft walls or obvious dynamic instability, simple PVP/PKP may not be able to form reliable bone cement–trabecular bone interlocking, and cement loosening, migration, recurrent collapse or kyphotic progression may still occur after surgery. Therefore, in recent years, a variety of improved technologies have been developed around improving the quality of bone cement distribution and enhancing the internal stability of the affected vertebra.

### 5.1 Bone cement distribution optimization and anti-migration techniques

Bone cement distribution optimization is the basis for improving minimally invasive treatment. Clinically, the distribution of bone cement can be extended from a single cleft cavity to the anterior column, middle column and posterior column regions of the vertebral body through high-viscosity bone cement, low-pressure injection, multi-point targeted puncture, bilateral approach, positional reduction and three-column augmentation. Unilateral bone cement multipoint anchoring PKP improves the stability of the affected vertebra from the perspective of puncture pathway and bone cement distribution, reflecting the concept of “multi-point distribution and local anchoring” (15). Three-column augmented PVP reduces the risks of cement loosening, migration and extrusion after augmentation by strengthening the multi-column structure of the vertebral body (16). Percutaneous pediculoplasty combined with vertebroplasty can extend the distribution of bone cement to the pedicle region, which may help strengthen the connection between vertebral body cement and posterior bony structures (17). Together, these methods show that the core of minimally invasive treatment of Kümmell disease is shifting from cleft filling to reconstruction of a stable mechanical structure.

### 5.2 Minimally invasive double-anchoring technique

Minimally invasive double-anchoring technique is one of the emerging composite stabilization strategies developed based on PKP and bone cement anchoring concepts. Its core is not simply to retain a bone cement tail in the pedicle, but combines “bone cement filling anchoring within the vertebral body” with “mechanical anchoring of bone cement screws in the pedicle region” to form a “screw–bone cement–vertebral body” composite stabilization construct. There is often a sclerotic bone wall or capsule-like tissue around the cleft of Kümmell disease. Conventional PVP/PKP bone cement is mostly concentrated in the necrotic cleft cavity, and it is difficult to achieve sufficient interlocking with cancellous bone. After repeated loading, it may loosen, migrate or collapse again. The new bone cement screw system combined with vertebroplasty can strengthen the mechanical connection between the cement mass and the pedicle region through screws, reducing the risk of cement migration (18); the new hollow pedicle screw combined with PKP also shows the potential to relieve pain, restore partial vertebral height and reduce bone cement migration (19).

This technique is suitable for stage I, stage II and partially reducible stage III cases with no obvious neurological compression, obvious intravertebral clefts, sclerotic cleft walls, bone cement aggregation or high risk of migration. Its advantage is that it retains the minimally invasive characteristics of PKP while enhancing anti-loosening, anti-rotation and anti-migration capabilities. It is especially suitable for patients whose stability provided by simple PVP/PKP is insufficient but who have not reached the need for open decompression and fixation. Its limitations should also be clear: for patients with obvious posterior wall disruption, severe spinal canal compromise, neurological dysfunction or rigid kyphotic deformity, this technique cannot replace posterior decompression, instrumented fusion or deformity correction and reconstruction; at present, the evidence is still mainly based on retrospective studies and preliminary biomechanical studies, and long-term therapeutic effects, indication boundaries and comparisons with mesh containers and instrumentation augmentation techniques still require further verification. Therefore, the minimally invasive double-anchoring technique should be regarded as a potential option for selected patients rather than a standard or superior treatment. Its clinical value still needs to be confirmed by multicenter prospective studies with larger sample sizes and longer follow-up.

### 5.3 Bone-filling mesh container and container-assisted techniques

Bone-filling mesh container and container-assisted techniques restrict the distribution direction of bone cement through controllable containers, allowing it to form a relatively regular supporting structure within the vertebral body, thereby reducing the risk of leakage into the paravertebral region, intervertebral disc and spinal canal. Compared with simple PVP/PKP, these techniques place more emphasis on “controlled filling” and “internal support”, and are suitable for patients with large cleft cavities, high leakage risk, remaining reduction potential and controllable posterior wall risk. Bone-filling mesh containers may have advantages in reducing bone cement leakage (14); mesh-hold bone-filling containers can improve cement filling quality and support morphology, and reduce fracture, migration and irregular distribution (20). However, the relevant evidence is mostly based on retrospective studies or case series, and the cost, indications and long-term maintenance of vertebral height still require further observation.

### 5.4 Intravertebral support devices

For cases with obvious vertebral collapse, insufficient anterior and middle column support, or difficulty maintaining height with simple bone cement augmentation, intravertebral support devices provide a new treatment direction. Minimally invasive transpedicular intravertebral cage technology can place an internal support structure through the transpedicular pathway to achieve internal reconstruction and height maintenance of the affected vertebra (21). Compared with traditional bone cement filling, this type of technology places more emphasis on mechanical support and internal structural reconstruction, representing the development trend of Kümmell disease treatment from bone cement filling toward internal support reconstruction. However, implant-related complications, long-term height maintenance effects, indication boundaries, and the advantages and disadvantages of combination with traditional instrumentation remain unclear. At present, it is more suitable as an exploratory or complementary strategy in selected cases.

## 6 Surgical treatment and complications of complex cases

For stage III Kümmell disease, especially those with posterior wall disruption, spinal canal compromise, obvious dynamic instability, neurological dysfunction or severe kyphotic deformity, it is often difficult to complete decompression, deformity correction and stabilization reconstruction with simple PVP, PKP or minimally invasive double-anchoring techniques at the same time. The treatment target of such cases should shift from simple vertebral augmentation to neural decompression, anterior and middle column support, posterior column stabilization and sagittal alignment restoration. Since most of the patients are elderly individuals with osteoporosis, the choice of surgical method needs to achieve a balance between fixation strength and surgical trauma.

Bone cement-augmented pedicle screws can improve the stability of the screw–bone interface. They are suitable for stage III cases with severe osteoporosis, kyphotic deformity or prominent vertebral instability, which helps to reduce the risks of screw loosening, pullout and instrumentation failure (22). Hollow pedicle screw anchoring with bone cement further forms a composite anchoring construct consisting of screws, bone cement and the affected vertebra, which can enhance anterior and middle column support and posterior instrumentation strength simultaneously. It has certain value for cases with posterior wall disruption but where stabilization reconstruction remains the primary goal (23). Short-segment and long-segment fixation should be selected according to bone quality, degree of collapse, kyphotic angle, spinal canal compromise and neurological function status. Short-segment fixation can reduce surgical trauma and fixation range, while long-segment fixation may have greater advantages in maintaining deformity correction and stress distribution (24). Recent Chinese studies have suggested that vertebral augmentation, bone grafting combined with short-segment pedicle screw fixation, and short-segment fixation combined with decompression after vertebral augmentation can be used as treatment options for patients with stage III disease or neurological compression symptoms (25, 26). For patients with significant spinal canal compromise, neurological deficits or rigid kyphotic deformity, decompression and fusion and osteotomy-based deformity correction remain irreplaceable, but the perioperative risks caused by advanced age, osteoporosis and underlying diseases should be fully evaluated (27).

Complication management is also important for complex cases of Kümmell disease. Bone cement leakage is related to posterior wall disruption, cleft morphology, cement viscosity, injection pressure and injection volume, and the risk should be reduced through preoperative risk stratification, low-pressure injection and fluoroscopic monitoring (9, 28). Cement loosening and migration are relatively specific complications of Kümmell disease and are often associated with sclerotic cleft walls, mass-like cement filling and pseudoarthrosis-like activity within the vertebral body (29). Other potential complications include recurrent vertebral collapse, adjacent vertebral fracture, infection, neurological injury, pulmonary cement embolism and instrumentation failure. Therefore, careful preoperative imaging evaluation, individualized surgical planning, strict intraoperative monitoring, anti-osteoporotic therapy and long-term follow-up are important for reducing the risk of postoperative failure.

## 7 Controversy, outlook and summary

At present, there are still three disputes about the diagnosis and treatment of Kümmell disease. First of all, the classification criteria have not been unified. The classical staging emphasizes the height loss of the vertebral body, the rupture of the posterior wall and the neural compression, which is of guiding value for clinical decision-making (8). However, the definition of dynamic instability, the degree of rupture of the posterior wall and neurological symptoms in different studies is inconsistent, which makes it difficult to directly compare the surgical method selection and the research results. In the future, we should integrate x-ray dynamic position, CT and MRI to establish a more unified classification system around the morphology of the intravertebral cleft, the integrity of the posterior wall, the spinal canal compromise, the reduction potential and the neurological status.

Secondly, the treatment boundary for patients with posterior wall rupture but no neural symptoms is still unclear. The traditional view is that posterior wall disruption increases the risk of bone cement leakage into the spinal canal and neurological injury, and therefore often favors combined fixation or decompression. However, some studies have shown that, with strict imaging evaluation, low-pressure cement injection and fluoroscopic monitoring, selected stage III patients without neurological symptoms may still achieve pain relief and functional improvement after PVP or PKP (5). Systematic evaluation suggests that vertebroplasty and posterior pedicle screw fixation combined vertebroplasty can be used for such patients. The former has more minimally invasive advantages in terms of operation time, bleeding volume and hospitalization time (30). Therefore, posterior wall disruption should not be regarded as an absolute contraindication to minimally invasive vertebral augmentation, nor should it be used as the sole criterion for selecting posterior fixation, decompression, or deformity correction. For stage III patients without neurological symptoms, treatment should be individualized according to the severity of posterior wall defect, the degree of spinal canal compromise, dynamic instability, reduction potential, risk of cement leakage or migration, and the patient's overall clinical condition. Selected reducible stage III cases without severe canal compromise may still be considered for vertebral augmentation or composite stabilization techniques under strict imaging evaluation and fluoroscopic monitoring, whereas patients with severe posterior wall defect, severe spinal canal compromise, neurological deficit, rigid kyphotic deformity, or poor reducibility should be managed as advanced or complex cases. This individualized decision-making pathway is summarized in the revised Figure 1.

Thirdly, the evidence level of new technologies such as minimally invasive double anchoring, bone-filled mesh bags, mesh-hold containers and vertebral support devices is still limited. These technologies aim to address bone cement clumping, loosening and migration, isolated filling of the cleft cavity and insufficient maintenance of vertebral height, reflecting a shift from simple cement filling toward composite stabilization (14, 18–21). However, most of the existing studies are small samples, single-center or retrospective studies, and the indications, follow-up time and efficacy indicators are not uniform.

This review has several limitations. First, although a structured literature search was performed, this article is a narrative review, and no formal risk-of-bias scoring was conducted. Second, the evidence for emerging techniques, including minimally invasive double-anchoring techniques, bone-filling mesh containers, mesh-hold containers, and intravertebral support devices, remains limited and is mainly derived from retrospective studies, small case series, or preliminary biomechanical studies. Accordingly, these techniques should be interpreted as potential options for selected patients rather than as standard or superior treatments, and their clinical value still needs to be confirmed by multicenter prospective studies with larger sample sizes and longer follow-up.

In general, the treatment of Kümmell disease should shift from simple pain relief toward classification-based decision-making, vertebral stability reconstruction and whole-process osteoporosis management. Stable cases should be managed mainly with anti-osteoporotic therapy and minimally invasive augmentation, unstable cases require cement distribution optimization and anchoring-based anti-migration strategies, whereas complex cases should be treated with fixation, decompression or deformity correction when necessary.
